# Evaluation of antimicrobial properties of nano-silver particles used in orthodontics fixed retainer composites: an experimental in-vitro study

**DOI:** 10.34172/joddd.2021.015

**Published:** 2021-05-05

**Authors:** Amirhossein Mirhashemi, Abbas Bahador, Ahmad Sodagar, Maryam Pourhajibagher, Ali Amiri, Elahe Gholamrezayi

**Affiliations:** ^1^Department of Orthodontics, Dentistry Faculty, Tehran university of Medical Sciences, Tehran, Iran; ^2^Department of Microbiology, Oral Microbiology Laboratory, School of Medicine, Tehran University of Medical Sciences, Tehran, Iran; ^3^Dental Research Center, Dentistry Research Institute, Tehran University of Medical Sciences, Tehran, Iran; ^4^Dentist, General Practitioner, Tehran, Iran

**Keywords:** Antibacterial properties, Fixed retainer, Growth inhibition zone, Silver nanoparticles

## Abstract

**Background.** The present study evaluated the antimicrobial efficacy of composite resins containing nano-silver (NAg) particles used in fixed orthodontic retainers.

**Methods.** Nano-composite resin samples with 1%, 2%, and 5% concentrations of NAg were prepared. The antimicrobial effectiveness of NAg was assessed against *Streptococcus mutans*, *Streptococcus sanguis*, and *Lactobacillus acidophilus* by the biofilm inhibition test (three-day-old biofilms), eluted components test (on days 3, 15, and 30), and disk-diffusion agar test after 48 hours. Measures of central tendency and index of dispersion were used to determine colony-forming units. Kruskal-Wallis test and Mann-Whitney U test were also used.

**Results.** The biofilm inhibition test showed a significant decrease in the colonies of *S. mutans* (87.64%, 96.47%, and 99.76% decrease), *S. sanguis* (98.13%, 99.47%, and 99.93% decrease), and *L. acidophilus* (81.59%, 90.90%, and 99.61% decrease) at 1%, 2%, and 5% concentrations of Nag, respectively, compared to the control groups. The colony-forming unit (CFU)/mL of tested microorganisms continuously decreased with increased NAg concentration. In the eluted component test, no significant differences were noted in the 3rd, 15th, and 30th days between the different concentrations of Nag-containing composite resin disks and control samples. According to the disk-diffusion agar test, there was no growth inhibition zone for the composite resin disks containing 1% and 2% concentrations of Nag. However, the growth inhibition zone was seen with a 5% concentration, with a diameter of 9.5±0.71 mm for *S. mutans*, 8.5±0.71 mm for *S. sanguis*, and 8±1.41 for *L. acidophilus*.

**Conclusion.** The incorporation of NAg into composite resins has antibacterial effects, possibly preventing dental caries around fixed orthodontic retainers.

## Introduction

One of the most challenging issues in orthodontics is the long-term stability of orthodontic treatments. The retention phase of orthodontic intervention is a crucial component of the treatment similar to the active phase. Post-orthodontic relapse (with varying degrees) occurs in 70%–90% of the cases.^1^ Many factors like age and maturity of patients, origin and the character of the anomaly, result of the orthodontic intervention, type of retainer, and compliance of the patients, can influence the relapse. Some studies have suggested different treatment plans that can enhance treatment stability. However, many orthodontists presume that the only way to obtain the desired alignment after debonding, especially in the lower anterior segment, is fixed bonded retainers, made of a piece of wire and composite resin bonded to teeth.^2-4^

The presence of appliances bonded to teeth with composite resin changes the self-cleansing contour of teeth, resulting in microbial plaque aggregation.^5,6^ Studies have shown that dental composite resins cause more bacterial plaque aggregation that contains more *Streptococcus mutans* than other restorative materials, such as amalgam or glass ionomer, or tooth structure.^7^

Fixed orthodontic appliances provide a unique environment, which can interact with cariogenic bacteria such as streptococci, causing bacterial colonization and plaque aggregation. Some research suggests that the level of cariogenic streptococci (number and volume) in dental plaque increases in response to fixed orthodontic appliances. However, after removing the appliances, it would regress to the normal level. The rough surface of orthodontic adhesive causes microorganism attachment and their rapid growth. Therefore, it is a major predisposing factor for demineralization.^8^ It has been shown that orthodontic appliances can alter oral conditions, such as increased plaque accumulation, decreased pH, and elevated *S. mutans* and *Lactobacillus acidophilus* colonization. It is important to know that of different pathogenic organisms present in plaque, *L. acidophilus* does not play a significant role in the initiation of the lesion but can enhance its progression.^9^ Studies have shown that just three weeks after banding and bonding in a patient with good oral hygiene, mature cariogenic plaque is detectable around the adhesive. In contrast, only primary plaque is present near the gingival margin.^10^

Nanotechnology manipulates materials at the nanometer level to use individual atoms and molecules to construct functional structures.^11^ In orthodontics, nanoparticles have been proposed for different purposes like caries inhibition,^12^ friction reduction,^13^ and increasing the bond strength.^14^ One of the accomplishments of nanotechnology is metal nanoparticles as potential antimicrobial agents. Silver is one of the common elements used in dental treatments. Silver has a higher antimicrobial effect than other metals. It has antimicrobial activity against many microorganisms in both metallic and ionic forms, and interestingly, even at a very low concentration, it is effective against biofilms. However, the antimicrobial action of silver is based on diffusion; therefore, a serious limitation of its use is limited reservoirs, resulting in the inability to create long-term effects.^15-17^ Since nanoparticles have more contact surfaces, small amounts of nano-silver (NAg) have antimicrobial effects like silver mass,^18,19^ but their ability to release high levels of ions at low concentrations distinguish them from other particles.^16^ Silver ion is very reactive and rapidly binds to negatively charged proteins, RNA, DNA, etc. This characteristic feature is the most critical part of the antibacterial mechanism.^20^

The present study aimed to determine the antimicrobial efficacy of NAg applied in the composite resins used in fixed orthodontic retainers.

## Methods

In this in vitro study, composite resin disks containing 1%, 2%, and 5% concentrations of NAg measuring 6 mm in diameter were used. Antimicrobial effects of disks against *S. mutans*, *S. sanguis*, and *L. acidophilus* were determined by disk-diffusion agar test, eluted components, and biofilm inhibition tests. In this study, 132 samples were assessed (72 samples in the eluted test, 24 in the disk-diffusion agar test, and 36 in the biofilm assay).

### Preparation of nano-particles

The NAg was prepared from silver nitrate. In this method, a silver nitrate solution (100 mg of silver nitrate in 20 mL of H_2_O) was stirred by a magnetic mixer for six hours and passed through nitrogen gas. The solution was irradiated by 20 kGy gamma-ray to prepare pure products with the homogeneous distribution of NAg particles, appropriate for medical and dental applications. The resulting deposit was heated at 40°C in an oven for five hours. After dehydration, the solution was converted to powder.^21^ Finally, scanning electron microscopy (SEM) and energy dispersive spectroscopy (EDAX) analyses were carried out on the NAg powder to determine particle size.

### Preparation of modified composite resin

To prepare dental composite resin containing 1%, 2%, and 5% (by weight) nanoparticles, respectively, 30, 60 of, and 150 mg NAg powder was mixed with 3000, 2940, and 2850 mg of Flow Tain (Reliance, USA) composite resin. Composite resin and nanoparticles were mixed in a semi-dark environment, using a glass slide as a mixing pad and a spatula until a uniform consistency was achieved.

### Composite resin disk preparation

**Figure 1:**
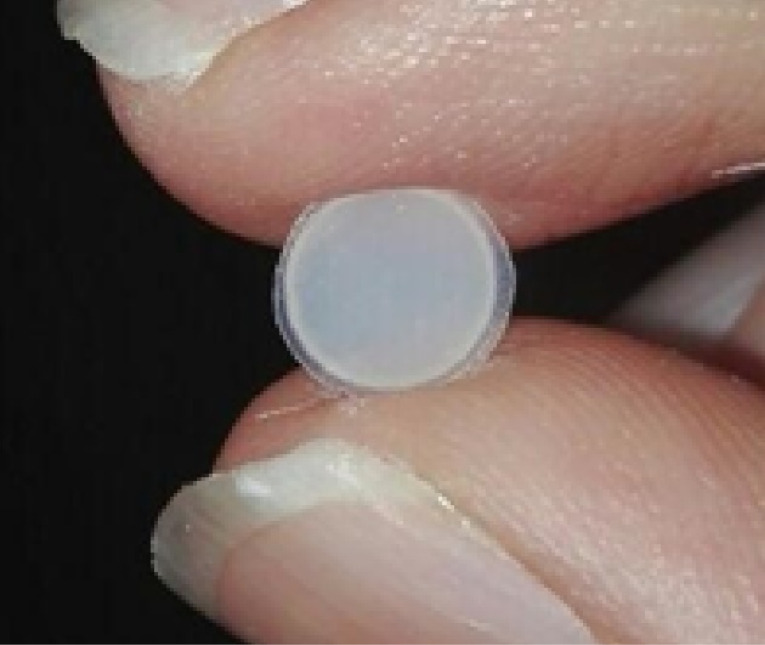
Composite resin disk.

Ring-shaped molds with a diameter of 5 mm were filled with composite resin, and to achieve a smooth surface, the molds were placed between two glass slabs. Visible light-curing (385 to 515 nm,1200 mW/cm^2^) was applied for 120 seconds (Bluephase® 16i, Ivoclar Vivadent AG, Australia) from each side (overall 240 seconds). After polishing, all the specimens (n=132) were sterilized in Iran’s Nuclear Science and Tech Center with gamma radiation (25 kGy) (Figure 1).

### Preparation of bacterial suspensions

To prepare bacterial suspensions, *S. mutans* (ATCC 25175), *S. sanguis* (ATCC 10556), and *L. acidophilus* (ATCC 4356) were used. All the bacteria were grown in brain-heart infusion broth (BHI; Difco, Sparks, MD, USA) at 37°C until the cells reached the mid-logarithmic phase (optical density=600 nm, 0.5 for *S. mutans* and *S. sanguis,* and 1 for *L. acidophilus*). *L. acidophilus* was grown under anaerobic conditions, and the two other bacteria were grown under aerobic conditions in the presence of 5% CO_2_.

Microorganism suspensions with 10^8^ colony-forming units (CFU)/mL were used to determine the antimicrobial effect of the composite resin containing NAg.

### Disk-diffusion agar test

The antimicrobial properties of NAg released from composite resin disks were studied through this test. The tested composite resin disks (n=24) were placed 2 cm apart on *brain heart infusion* (BHI) agar plates inoculated with a bacterial solution (~1.5×10^8^ CFU/mL) by a sterile swab. The diameter of the inhibition zone was measured by a ruler after a 48-hour incubation at 37°C.^22^

### Biofilm inhibition

Three-day-old biofilms of each bacterium were produced on composite resin disks (n=36) in 24-well plates. Each well was inoculated with adjusted bacterial inoculum. The biofilm was grown at 37°C (2 mL of BHI containing 0.5% sucrose). To eliminate planktonic and loosely attached bacteria, each disk was rinsed with phosphate-buffered saline solution (PBS, pH=7.0) after 72 hours. All the specimens were sonicated at 50 Hz frequency in sterile saline solution for five minutes and vortexed in maximum power for one minute to count the CFUs responsible for biofilm formation. The CFU/mL of the microorganism present in the suspension was counted with the drop-plate method using serial dilution in microtiter plates.^22^

### Eluted components test

Antibacterial activities of possible eluted components of NAg in composite resin disks (n=72) were also evaluated. Then, they were placed in tubes containing 1 mL of BHI media. On days 3, 15, and 30, 50-µL volumes of the media were removed from each test tube and poured into new tubes, and 50 μL of bacterial culture with 10^6^ CFU/mL were also added to them. Later, the tubes were shaken at 300 rpm at 37°C for 24 hours. The CFU/mL of the microorganism in the suspension was counted as described above. At this time, the specimens were incubated at 37°C and a pH of 6.5‒7.5 in a dark environment.^22^

All the three tests (disk-diffusion agar, biofilm inhibition, and eluted components tests) were repeated three times.

### Statistical analysis

SPSS 21 (IBM, Chicago) was used to analyze the data. Measures of central tendency and index of dispersion were used to determine colony-forming units in the biofilm inhibition and eluted components tests. Besides, the diameters of the growth inhibition zone in the disk-diffusion agar test for different concentrations of silver nanoparticles were reported. Since the data did not exhibit normal distribution, the Kruskal-Wallis test was used to analyze colony-forming units and growth inhibition zones at different concentrations of silver nanoparticles. Considering the significance of the Kruskal-Wallis test results in the biofilm inhibition test, two-by-two comparisons were made at different nanoparticle concentrations using the Mann-Whitney U test. A *P* value of < 0.05 was considered statistically significant.

## Results

### Biofilm inhibition test

Mature biofilms on Nag-containing composite resin disks and the control group were assessed. Kruskal-Wallis test revealed significant differences in the number of colonies of *S. mutans* (*P* = 0.015), *S. sanguis*(*P* = 0.016), and *L. acidophilus* (*P* = 0.016) in composite resin disks containing NAg (1%, 2%, and 5%), compared to the control group. Colony counts in Nag-containing composite resin disks were significantly lower than the control group. Mann-Whitney U test showed significant differences between different NAg concentration groups (0% and 1%, 0% and 2%, 0% and 5%, 1% and 2%, 1% and 5%, and 2% and 5%) and the number of colonies in each species (*P* = 0.05). Furthermore, an increase in the concentration of NAg particles in composite resin disks decreased the colony counts. The results are presented in Table 1.

**Table 1:** Results of biofilm inhibition tests for *S. mutans*, *S. sanguis*, and *L. acidophilus* for composite disks containing NAg (1%, 2%, and 5%) and control group

**Group**	**Microorganism**	**Mean ± SD**	**CFU/mL decrease (%) compared to the control group**
Control	*S. mutans*	56666±30550	-
	*S. sanguis*	446666±117189	-
	*L. acidophilus*	146666±32145	-
Composite disks containing 1% nanoparticle	*S. mutans*	7000±1000	87.64
	*S. sanguis*	8333±1527	98.13
	*L. acidophilus*	27000±7549	81.59
Composite disks containing 2% nanoparticle	*S. mutans*	2000±1000	96.47
	*S. sanguis*	2333±1527	99.47
	*L. acidophilus*	13333±2516	90.9
Composite disks containing 5% nanoparticle	*S. mutans*	133±57	99.76
	*S. sanguis*	300±100	99.93
	*L. acidophilus*	566±251	99.61

In the 1% NAg group, CFU/mL reduction was observed as follows: 87.64% in *S. mutans*, 98.13% in *S. sanguis*, and 81.59% in *L. acidophilus* compared to the control group. Similarly, in 2% and 5% Nag groups, there were reductions of 96.47% and 99.76% in *S. mutans* CFU/mL, respectively, reductions of 99.47% and 99.93% in *S. sanguis*CFU/mL, respectively, and reductions of 90.90% and 99.61% in *L. acidophilus* CFU/mL, compared to the control group.

### Disk-diffusion agar test

**Figure 2:**
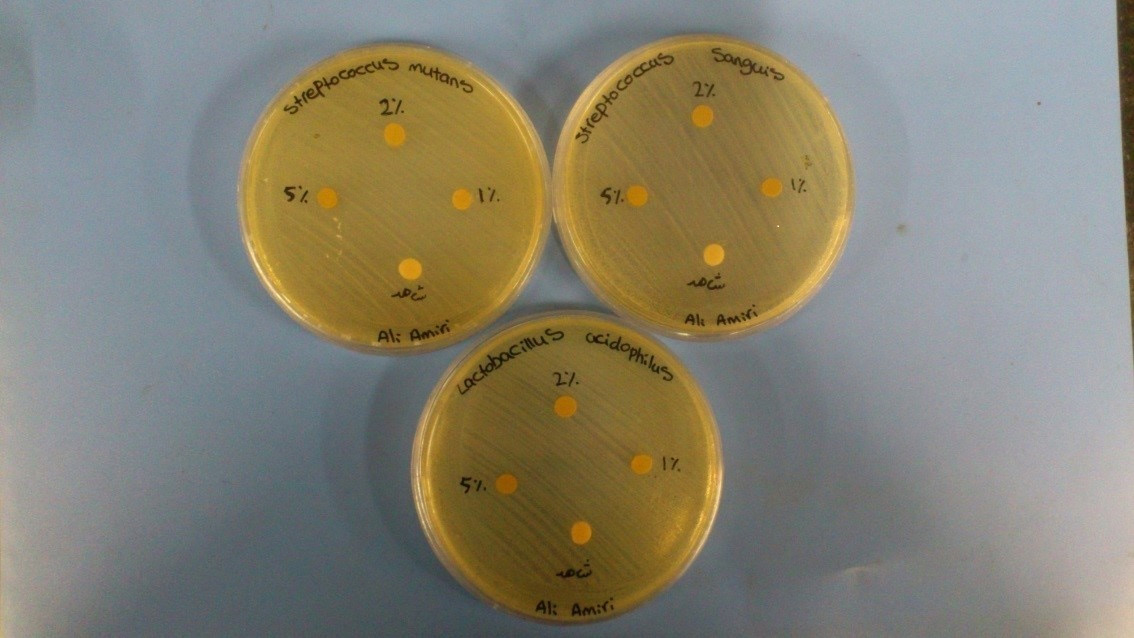
Disk-diffusion agar test.

According to the agar diffusion test, there was no growth inhibition zone around Nag-containing composite resin disks with 1% and 2% concentrations; however, growth inhibition zone was seen in 5% concentration, with a diameter of 9.5±0.7 mm for *S. mutans*, 8.5±0.7 mm for *S. sanguis*, and 8±1.4 for *L. acidophilus* (Figure 2).

### Eluted components test

According to the Kruskal-Wallis test, for all the three bacteria (*S. mutans*, *S. sanguis*, and *L. acidophilus*), no significant differences were observed on days 3, 15, and 30 between the composite resin disks containing different concentrations of NAg compared with control specimens (*P* > 0.05).

## Discussion

The retention phase is required to prevent post-orthodontic relapse. Nowadays, many orthodontics choose bonded fixed retainers made of a piece of wire and bonded to the teeth with composite resin. They include either conventional restorative or specific orthodontic bonding resins.^23,24^ Regrettably, composite resins do not display any antibacterial effects after curing. Moreover, composite resins cause dental plaque accumulation in the long term compared to enamel and other restorative dental materials. Cariogenic bacteria such as *S. mutans*can readily grow on the composite resin surface, resulting in plaque accumulation that often leads to acid production and causes demineralization and gingival problems.^25,26^

Other materials, such as glass ionomer cements (GICs) and resin-modified glass ionomer cements (RMGICs), exhibit antibacterial effects. They release fluoride, which interferes with the growth or metabolism of cariogenic bacteria and causes lower *S. mutans* accumulation. Furthermore, they promote remineralization. Unfortunately, the mechanical properties of these materials are considerably lower than composite resin.^27,28^ Baysal et al,^28^ in an experimental study, evaluated the shear bond strength (SBS), fracture mode, and wire pull-out (WPO) resistance between RMGIC and conventional orthodontic composite resins used as a lingual retainer adhesive. They reported that the SBS and WPO values of RMGIC were significantly lower than conventional orthodontic composite resin, with no difference in the fracture mode. For these reasons, composite resin remains the most common material used with fixed retainers.

Currently, various particles are incorporated into adhesive resins to induce antimicrobial characteristics. Attempts to add antimicrobial agents, such as fluoride and chlorhexidine to composite resins, showed questionable antimicrobial efficiency; furthermore, they can negatively affect mechanical properties.^29-32^ On the other hand, silver is widely used as an antibacterial agent. The advantages of silver are: (1) biocompatibility with human cells; (2) continuous ion release and thus long-run antimicrobial effects; (3) low bacterial resistance compared to antibiotics.^33^ Many studies have shown the antibacterial effects of NAg particles.^19,34,35^ Different properties of nanoparticles, such as size, shape, consistency of particles, and surfactant types, are exclusive.^36^ These properties can affect the antimicrobial efficacy of nanoparticles.^37^

The exact mechanism of the antibacterial effect of NAg is not clear yet, but it seems that structural and morphological alterations are part of the mechanism. As silver ion enhances electron displacement, it can play a catalytic role in converting oxygen into reactive oxygen species, leading to bacterial structural damage, such as protein and enzyme denaturation, inactivating bacteria. Silver ions also affect the three essential components of bacterial cells, including bacterial DNA, peptidoglycan cell wall, and plasma membranes, inducing bactericidal effects.^38,39^ Nanoparticles have more impressive contacts with microorganisms due to their large surface area; this feature improves their antimicrobial properties.^40^

Biofilm is a thick accumulation of microorganisms attached to an area. Biofilms exhibit increased drug tolerance and are four times more resistant than planktonic forms. Oral biofilms are a virulence factor in many oral infectious diseases, such as periodontitis, endodontic infections, and dental caries, and can accelerate dental caries progression.^17,41^ In the present study, according to the biofilm inhibition test, colony counts in Nag-containing composite disks were significantly lower than the control group. The antimicrobial effects of orthodontic base plates containing Nag were evaluated in a clinical trial. Biofilm inhibition analysis demonstrated that orthodontic base plates containing NAg inhibited biofilms of all the bacteria tested (*S. mutans*,* S. sobrinus*,* L. acidophilus*,and* L. casei*) by 20.1%‒79.9% compared to the control group.^42^ Another study showed that NAg in bonding agents considerably reduced biofilm metabolic activity, CFU, acid production, and gene expressions, with no adverse effects on microtensile dentin bond strength and cytotoxicity. A primer containing NAg also showed antimicrobial effects on *S. mutans* far from the surface in a culture medium. These findings suggest ion release from a resin containing NAg, which leads to long-distance killing ability.^43^

In the present study, the antimicrobial effect of NAg was proportional to NAg concentration; an increase in the percentage of NAg particles in composite resin disks decreased colony counts. Ghahremanloo and Movahedzadeh^40^ evaluated antimicrobial effects of acrylic resins containing NAg against *Candida albicans* and *S. mutans*. They concluded that an increase in NAg concentration in acrylic resins resulted in more significant antimicrobial effects. Azarsina et al^44^ concluded that the incorporation of NAg into composite resin reduced *S. mutans* and *Lactobacillus* colony counts; higher concentrations (1% in comparison of 0.5% by weight) resulted in better antibacterial properties, consistent with the present study.

In the present study, according to the agar diffusion test, there was no growth inhibition zone for the composite disks containing NAg particles at 1% and 2% concentrations; however, the growth inhibition zone was seen with 5% concentration. Cheng et al^33^ investigated the antibacterial effects of NAg-containing primers impregnated into human dentin blocks against *S. mutans*. In the agar diffusion test, the primer containing 0.1% NAg increased the *S. mutans* inhibition zone by 6.5-fold, compared to the commercial control primer. As mentioned above, in the present study, there was no inhibition zone with 1% and 2% concentrations of NAg. Perhaps, the factor that causes this difference is the potential antibacterial functions of the primer.^33^ Also, the specific properties of nanoparticles (size, shape, etc.) affect antibacterial effects.

It should be noted that the incorporation of nanoparticles into composite resin can affect its mechanical properties. Some previous studies have reported that incorporating silver compounds into dental materials at concentrations of ≥10% significantly reduces compressive strength, elastic modulus, and tensile strength.^45,46^ Akhavan et al^47^ evaluated the effect of incorporating silver and hydroxyapatite nanoparticles on the SBS of orthodontic adhesives. They showed that incorporating 1% and 5% silver/hydroxyapatite nanoparticles increases and maintained the SBS of orthodontic adhesives, respectively, while undesirable effects were seen at 10% concentration. Another study showed that incorporating NAg particles to orthodontic primer affected neither the SBS nor tensile strength of polymethyl methacrylate. They showed that the tensile strength of polymethyl methacrylate specimens containing NAg decreased compared to the control group.^48^ In general, it appears that NAg particles can affect various properties of composite resins, which likely depends on the concentration and size of nanoparticles.

Overall, it seems that NAg particles, added to orthodontic fixed retainer composite resins, exhibit desirable antibacterial effects. However, due to the specific conditions of the oral cavity (presence of saliva, thermal changes, food with different pH, etc.), the generalization of this experimental finding to clinical conditions is limited. More studies are required to determine the efficacy of NAg-containing composite resins on clinical conditions.

## Conclusion

1. NAg-containing composites induced antibacterial activity.
2. Increasing the concentration of NAg led to more antibacterial effects.
3. NAg-containing composite resins significantly reduced *S. mutans*, *S. sanguis,*and *L. acidophilus* colony counts.
4. No growth inhibition zone was seen at 1% and 2% concentrations of NAg, but it was seen with 5% concentration.

## Authors’ Contributions

AM and AS conceptualized and administered the study. AB, AA, and MP performed laboratory tests. EG prepared and submitted the manuscript. All authors have read and approved the final manuscript.

## Funding

Self-funded.

## Competing Interests

The authors declare no conflict(s) of interest related to the publication of this work.

## Ethical Approval

This study was registered and approved under the code 8911272002 in the Faculty of Dentistry, Tehran University of Medical Sciences.
